# Novel Polymerization of Dental Composites Using Near-Infrared-Induced Internal Upconversion Blue Luminescence

**DOI:** 10.3390/polym13244304

**Published:** 2021-12-09

**Authors:** Shu-Fen Chuang, Chu-Chun Liao, Jui-Che Lin, Yu-Cheng Chou, Tsung-Lin Lee, Ting-Wen Lai

**Affiliations:** 1 School of Dentistry and Institute of Oral Medicine, National Cheng Kung University, No.1 Universal Road, Tainan 70101, Taiwan; liao2300244@hotmail.com (C.-C.L.); bear889028@gmail.com (Y.-C.C.); finrebecca@gmail.com (T.-W.L.); 2 Department of Stomatology, National Cheng Kung University Hospital, 138 ShengLi Road, Tainan 70403, Taiwan; a_apple_permanent@hotmail.com; 3 Department of Chemical Engineering, National Cheng Kung University, No.1 Universal Road, Tainan 70101, Taiwan; jclin@mail.ncku.edu.tw

**Keywords:** resin composite, polymerization, upconversion, near-infrared, microhardness

## Abstract

Blue light (BL) curing on dental resin composites results in gradient polymerization. By incorporating upconversion phosphors (UP) in resin composites, near-infrared (NIR) irradiation may activate internal blue emission and a polymerization reaction. This study was aimed to evaluate the competency of the NIR-to-BL upconversion luminance in polymerizing dental composites and to assess the appropriate UP content and curing protocol. NaYF_4_ (Yb^3+^/Tm^3+^ co-doped) powder exhibiting 476-nm blue emission under 980-nm NIR was adapted and ball-milled for 4–8 h to obtain different particles. The bare particles were assessed for their emission intensities, and also added into a base composite Z100 (3M EPSE) to evaluate their ability in enhancing polymerization under NIR irradiation. Experimental composites were prepared by dispensing the selected powder and Z100 at different ratios (0, 5, 10 wt% UP). These composites were irradiated under different protocols (BL, NIR, or their combinations), and the microhardness at the irradiated surface and different depths were determined. The results showed that unground UP (d50 = 1.9 μm) exhibited the highest luminescence, while the incorporation of 0.4-μm particles obtained the highest microhardness. The combined 20-s BL and 20–120-s NIR significantly increased the microhardness on the surface and internal depths compared to BL correspondents. The 5% UP effectively enhanced the microhardness under 80-s NIR irradiation but was surpassed by 10% UP with longer NIR irradiation. The combined BL-NIR curing could be an effective approach to polymerize dental composites, while the intensity of upconversion luminescence was related to specific UP particle size and content. Incorporation of 5–10% UP facilitates NIR upconversion polymerization on dental composites.

## 1. Introduction

Photopolymerization of resin-base composites (RBCs) with blue lights (BL) was introduced in the 1980s [1]. BL irradiation activates the photosensitive initiator camphorquinone to react with a tertiary amine accelerator and generate primary free radicals. These radicals react with the dimethacrylate monomer and break the C=C double bonds to start a crosslinking reaction [2]. This process denoted a great advance for restorative dentistry since the operators have sufficient time to manipulate the RBC materials and solidify them on demand. Despite the convenience brought about by BL polymerization, dentists encounter problems related to insufficient curing depths [3]. The BL irradiance significantly attenuates in the composite materials to cause gradient polymerization, which means that the degree of conversion (DC) decreases with the depth of cavities [4]. Insufficient DC leads to poor mechanical and biological properties, such as compromised stiffness and wear resistance [5,6], increased solubility and water adsorption [7], and tissue irritation related to monomer release [8,9]. Additionally, incomplete polymerization hinders the construction of tooth–resin adhesion at the cavity floors [10,11] and impairs the durability of restorations. Clinicians usually place RBCs incrementally or use high-intensity lights to ensure adequate polymerization. However, detrimental effects of time consumption, air entrapment during placing increments [12], and raised shrinkage stress are associated with these techniques [13,14]. To date, complete and homogeneous polymerization of RBC restorations remains a goal to accomplish.

The near-infrared (NIR) spectrum normally covers a wavelength range from 750 nm to 2500 nm. In dental RBCs, NIR exhibits a lower scattering coefficient compared to BL and may transmit deeply [15]. Since the late 2000s, the NIR-to-visible upconversion curing technique has been undertaken to obtain bulk polymerization of methyl methacrylate [16,17]. Upconversion describes an optical process in which the sequential absorption of photons leads to the emission at a wavelength shorter than the excitation wavelength. The advanced upconversion phosphors (UP) materials such as NaYF_4_: Tm/Yb and NaYF4: Er/Yb, and YVO_4_ may convert NIR to visible light via the energy pathway of either a single-photon Stokes process [18] or two-photon upconversion (anti-Stokes process) [19]. The doped lanthanide ions (Y^3+^, Tm^3+^, and Yb^3+^) have a multitude of electronic energy states, and the phonon-assistance cooperative energy transfer between these ions facilitates the occurrence of upconversion [20,21]. The Yb^3+^ ion usually works as a sensitizer to absorb energy and actively transfers to other codopants with fluorescence resonance. Tm^3+^ works as an activator to provide pumped protons. The sequential absorption of two photons recreates the excited state. Then, a second upconversion occurs as the emission of visible light. The host material also plays an important role in the luminescent efficiency and emission profile [22]. Among the UP materials, Tm^3+^/Yb^3+^ co-doped NaYF_4_ is recognized to be excited by 980-nm NIR with low thresholds and to emit blue light efficiently.

The application of an NIR-upconversion reaction for the purpose of dental composite polymerization was initiated by Uo et al. [15], in which UP powder (Y_2_O_3_: Er/Tm/Tb) was added into dimethacrylate monomers to serve as both reinforcing fillers and an upconverter to polymerize resin. Though the NIR excitation induced the blue luminescence with the prepared composites, the obtained hardness value was inferior to the commercial product. In another study, by Stepuk et al. [23], the experimental UP-containing composites could be solidified up to 7 mm deep under NIR irradiation. With these findings, the NIR upconversion curing method could be a promising approach in polymerizing RBCs. NIR transmits through the resin and tooth substances more deeply than BL does, and the glowing of blue luminescence inside the restorations supposedly facilitates homogeneous polymerization that conventional curing does not reach. However, the mechanical properties of polymerized composites through this approach should be examined if they meet the clinical demands.

To apply the NIR upconversion chemistry on polymerizing RBCs, more realizations in the UP particles and their photoluminescence properties are mandatory Since the previous study showed that the UP-loaded resin exhibited low hardness and stiffness [15], an alternative approach by using UP as adjunct fillers rather than constituent fillers is anticipated to provide adequate mechanical properties and enhanced polymerization. Accordingly, this study would examine the validity and efficacy of the NIR-to-BL upconversion luminescence in curing UP-modified composites. The null hypothesis was that the new curing method facilitated comparable polymerization of RBC materials to that of conventional BL curing.

## 2. Materials and Methods

### 2.1. UP Materials’ Preparation and Characterization

NaYF_4_ (Yb^3+^/Tm^3+^ co-doped) powder (BL-IR-411 Blue, NCC, Taipei, Taiwan) was used as the NIR upconversion material. The UP powder was dispersed in alcohol, ultrasonically vibrated, and then harvested using copper grids. The morphology and atomic composition of UP particles were examined under a transmission electron microscope (TEM) (JEM-2010 Electron Microscope, JEOL, Tokyo, Japan) and the equipped energy dispersive spectroscopy (EDS).

In order to prepare UP particles of different sizes, 10-g UP were ground with a ZrO_2_ ball (3 mm in diameter, 450 g) in deionized water for either 4, 6, or 8 h in a ball-milling machine (Benchtop Ball Mill, BMT-30D, Hsiangtai, Taiwan). The particle size and distributions of the unground and ground powders were determined by a dynamic light scattering (DLS) method using a Delsa™ Nano C particle analyzer (Beckman Coulter, Fullerton, CA, USA).

### 2.2. NIR Irradiation Source and Characterization of Upconversion Emission

A continuous-wave diode laser (980D2000, Tanyu, Kaohsiung, Taiwan) with adjustable irradiance output provided the NIR excitation light source (λ = 980 ± 5 nm). The maximal radiant power output was 2111 mW. The beam size was 3 mm in diameter, and was defocused to a spot size of 4 × 5 mm^2^ by an attached aperture at a working distance of 10 cm. To characterize the source NIR, the high-intensity NIR was first attenuated through a 1/10 beam splitter and then examined with a full-spectrum fluorescence spectrometer (HR4000CG-UV-NIR, Ocean Optics, Dunedin, FL, USA). Afterward, the original intensity was reconverted by a 10× amplification.

**Figure 1:**
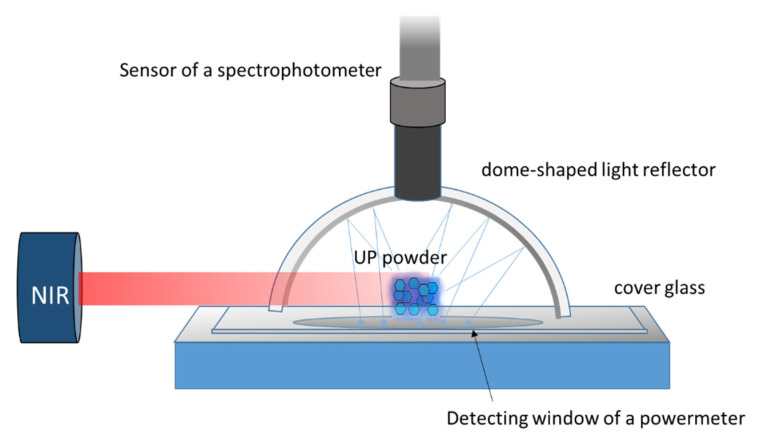
The device designed to measure the emitted luminous intensity of bare particles.

The emitted luminescence spectra and intensities were measured using a method referred to by Ivanova et al. [24] (Figure 1). Different amounts (0.005–0.25 g) of unground UP powder were packed into stacks (1–5 mm wide, 1–5 mm long, and 1–2 mm high). Each stack was placed above the sensor window of a powermeter (TEO, Nova II, Taipei, Taiwan) and covered with a dome-shaped light reflector. NIR was projected on the sample through a small aperture and activated the emitted lights. The spectrometer and powermeter detected the spectra and intensity of excited emission from the top and bottom of specimens, respectively. The irradiances of the emitted blue luminescence (410–500 nm) and NIR (750–850 nm) were derived by the total power output and their ratios on the spectra. The relative intensity of blue emission under various levels of NIR power (74–2111 mW) was determined by measuring the luminous intensity of 0.025-g UP particles. For each amount or NIR power, triple measurements of output intensity were performed.

### 2.3. Effects of UP Particle Size

The effects of UP particle sizes were first evaluated in regards to the emission intensity of bare particles. Then, 0.025 g of as-obtained and milled (after 4, 6, and 8 h) UP particles was packed into stacks respectively, and their luminous intensity under excitation NIR (2000 mW) was assessed, as mentioned.

**Figure 2:**
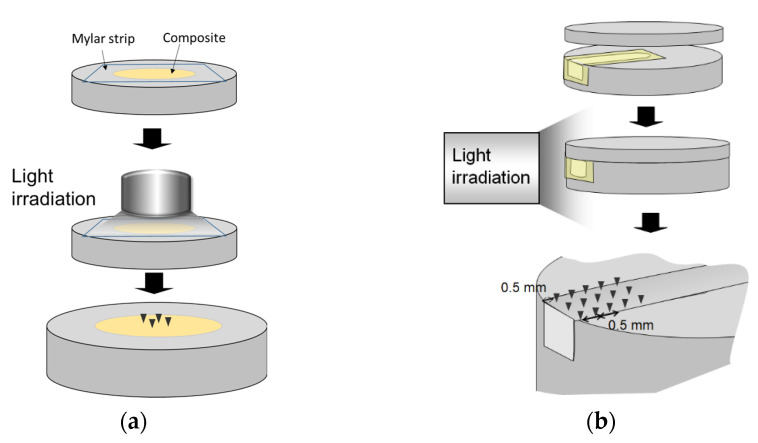
(**a**) Illustration of the microindentation test on the surface of a polymerized composite. (**b**) The experimental setup for the depth profiles of microhardness inside the composite specimens.

To select appropriate UP particles, their abilities to polymerize the RBCs were also examined. In a preliminary study, the experimental composites were prepared by mixing the UP particles into the resin adhesive (Scotchbond Multi-purpose adhesive, 3M ESPE) at 50 wt%. However, this experimental composite was not hardened enough even after 10-min NIR irradiation. Alternatively, the UP powders were separately mixed with a commercial microhybrid composite (Filtek Z100, 3M ESPE, St. Paul, MN, USA) at the ratio of 5 wt%. Each prepared composite was filled into a cylindrical cavity (4 mm in diameter and 2 mm deep) on a steel mold and covered with a Mylar strip to prevent the formation of an oxygen-inhibited layer (Figure 2a). The composite was irradiated with NIR for 5 min. The hardness on the top surface was examined using a microindentation tester (HMV-2, Shimadzu, Japan) with a Knoop indenter under a 25-g load and 20-s dwell time (*n* = 10). All the measurements were performed in a darkroom with a dim red light. For each kind of particle, nine measurements were performed.

### 2.4. Effects of UP Content and Irradiation Protocol on Surface Microhardness

UP powders of the selected size were premixed with an alcohol solution of silane methacrylate (Monobond-S, Ivoclar Vivadent, Schaan, Liechtenstein) for 10 min, and the excessive silane agent was discarded. The treated powder was mixed with a base composite Filtek Z100 at different ratios to generate three composites, Z100 (0% UP), UP5 (5 wt% UP), and UP10 (10 wt% UP), by hand-dispensing and then placed in a vacuum ball for 24 h to allow the evaporation of alcohol solvent. Afterward, each composite was placed into the circular metal mold and irradiated under different irradiation protocols as:

BL: irradiation with a LED curing light (SmartLite, Dentsply Sinora, Konstanz, Germany) with 950-mW/cm^2^ irradiance for 20–140 s;

NIR: irradiation with 20-s to 140-s NIR;

BL+NIR: irradiation by a BL LED for 20 s first, followed by 0–120-s NIR irradiation.

The microhardness on the irradiated surface of prepared composites was examined, as stated earlier. For each curing condition, nine microindentations on three samples were executed.

### 2.5. Depth Profiles of Microhardness

To examine the depth profile of polymerization, a method modified from Versluis et al. [25] was adapted (Figure 2b). Z100, UP5, and UP10 were separately filled into a rectangular slot (2.5 mm × 3 mm × 20 mm) on a steel mold, covered with a Mylar strip and a metal lid but leaving a side window. The composite was irradiated through the window with either 20-s, 80-s, or 140-s BL curing, or a combined protocol, consisting of 20-s BL plus 60-s or 120-s NIR. After irradiation, the metal lid was removed to expose the top surface. The microindentation tests were performed on the irradiated sides, and the top surfaces by placing indentations every 0.5 mm from the edge. At each depth, three measurements were taken in the middle of the slot and at 0.3 mm away. The examinations were repeated three times (*n* = 9) for each group.

### 2.6. Statistical Analysis

A one-way analysis of variance test was conducted to examine the statistical differences among the experimental groups, followed by post hoc Sheffe tests using SPSS 17.0 software (SPSS, Chicago, IL, USA). A *p*-value < 0.05 indicated the statistical significance.

## 3. Results

### 3.1. Characterization of the UP Materials

**Figure 3:**
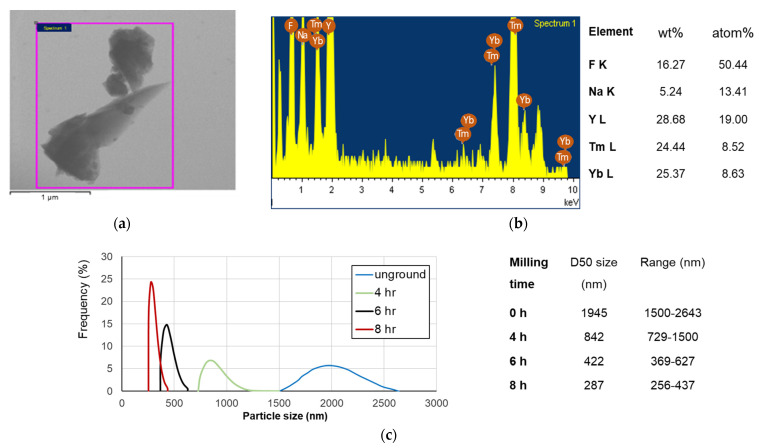
(**a**) A TEM image of as-obtained UP particles. (**b**) EDS analytic result and the element compositions of the UP particles. (**c**) UP particle size distribution after different ball-milling times.

TEM-EDS analysis revealed the irregular morphology of UP particles and the chemical compositions consisting of F, Na, Y, Tm, and Yb (Figure 3a,b). The DLS measurements of unground particles showed a size range from 1500 to 2643 nm with a median size (d50) of 1945 nm. For the particles receiving 4, 6, and 8 h ball milling, their d50 sizes and ranges were 842 nm (729–1500 nm), 422 nm (369–627 nm), and 287 nm (256–437 nm), respectively (Figure 3c).

### 3.2. Upconversion Luminescence Intensity and Spectrum

**Figure 4:**
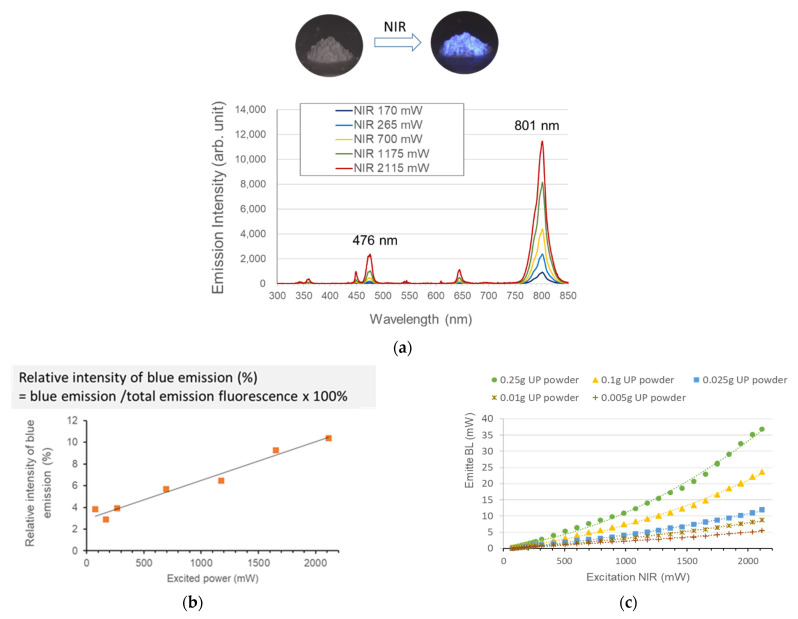
(**a**) NIR excited UP spectrum. (**b**) Relative intensity ratios representing the percentages of BL emission to the total upconversion luminescence. (**c**) Luminous intensities from different amounts of UP particles.

The spectrum of the upconversion luminescence indicated the presences of one strong NIR peak at 801.6 nm, two blue emissions at 450 nm and 476.4 nm, one weak red emission at 646 nm, and other faint luminescence (Figure 4a). The measurement revealed that NIR should exceed 74 mW to obtain detectable emission luminescence. When the incident powers changed from 74 to 2111 mW, the intensity of the emitted blue luminescence increased in a non-linear relationship to the excitation power. The relative intensity ratio, as the percentages (%) of BL emission to the total upconversion luminescence, was not constant but amplified with the NIR power (Figure 4b).

The emitting-excitation plots of UP in different amounts (0.005–0.25 g) are illustrated in Figure 4c. A regression analysis indicated a quadratic dependence of the emission intensity on both the UP amount and the excited NIR power.

### 3.3. Effect of Particle Size on Luminescence Intensity and Composite Polymerization

**Figure 5:**
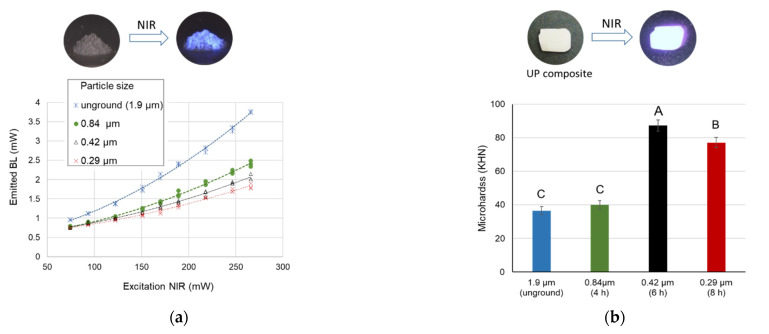
(**a**) Luminous intensities from UP particles of different sizes. (**b**) Microhardness of polymerized composites containing UP particles of different sizes. Different uppercase letters indicate significant differences (*p* < 0.05) among groups.

All the unground and ground particles glowed upconversion luminescence under NIR, and the emitted intensity increased with the incident irradiances. With the same irradiance, unground UP powder presented the strongest blue luminescence (Figure 5a).

The surface microhardness test on these composites blended with UP particles revealed a different result. The composite containing 0.42-μm (6-h milling) particles exhibited the highest microhardness, followed by those of 0.29-μm (8-h milling), 0.84-μm (4-h milling), and unground particles (Figure 5b). Accordingly, the 0.42-μm UP powder was chosen to prepare the experimental composites for the subsequent experiments.

### 3.4. Effects of UP Content and Irradiation Protocol on Surface Microhardness

**Figure 6:**
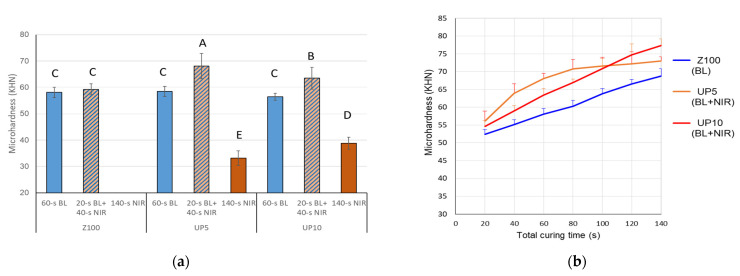
(**a**) Surface microhardness on Z100 and UP-containing composites irradiated by different protocols. Different uppercase letters indicate significant differences (*p* < 0.05) among groups. (**b**) Surface microhardness of Z100 and UP-containing composites with a total curing time from 20 to 140 s. Z100 was cured with BL. UP5 and UP10 were cured with 20-s BL, followed by NIR irradiation from 0 to 120 s.

The surface microhardness of three composites (Z100, UP5, and UP10) are illustrated in Figure 6a. The 60-s conventional BL curing resulted in comparable microhardness values (56.46–58.09 KHN) in three composites. Under a combined curing protocol (20-s BL and 40-s NIR), UP5 and UP10 presented significantly higher values (68.06 and 63.46 KHN, respectively). Sole NIR irradiation on UP5 and UP10, even extending to 140 s, only achieved mean hardness values of 33.15 and 38.78 KHN. Accordingly, sole NIR irradiation was not adapted in the following experiments.

Three composites were also irradiated with either BL or combined BL-NIR irradiations sequentially from 20 to 140 s, and then received the surface microindentation tests. Both UP composites attained significantly higher microhardness compared to their correspondent Z100 at each time point (Figure 6b). UP10 showed continuous growth in microhardness along the NIR irradiation duration, but UP5 showed a bounded exponential plot. As a result, UP5 exhibited the highest microhardness with 80-s NIR irradiation, but was surpassed by UP10 when NIR irradiation exceeded 100 s.

### 3.5. Depth Profiles of Microhardness

**Figure 7:**
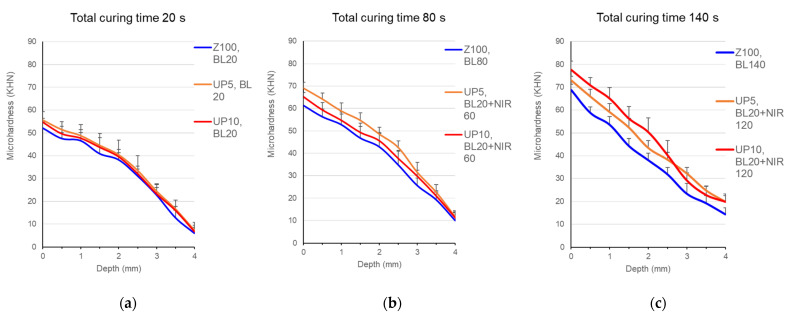
Microhardness at the depth profiles of Z100 and UP-containing composites after irradiation of (**a**) 20 s, (**b**) 80 s, and (**c**) 140 s.

Under 20-s BL irradiation, all the composites, Z100, UP5, and UP10, showed comparable microhardness on the irradiated surface and similar declinations along the depth (Figure 7a). For the curing courses of 80 and 140 s, Z100 was irradiated by BL while UP5 and UP10 received an initial 20-s BL curing and a second NIR irradiation. With 80-s curing, UP5 presented significantly higher microhardness than UP10 and Z100 for the top 3 mm (*p* < 0.05) (Figure 7b). Contrarily, UP10 showed superiority compared to the other two after 140-s irradiation (*p* < 0.05) (Figure 7c). Both UP composites did not differ within the depths below 2.5 mm.

## 4. Discussion

With advances in photochemistry, the NIR-excited materials received much attention for various applications in biomedical applications including bioimaging and phototherapy. The upconversion material used in this study was Yb^3+^/Tm^3+^-doped NaYF_4_, which is particularly favorable in the biomedical applications due to its capability to enable high-resolution, in-depth imaging and low toxicity [26,27]. Moreover, the versatile synthetic strategy of this material reduces the costs and makes its uses more plausible.

The upconversion process of NaYF_4_: Yb^3+^/Tm^3+^ is based on the sequential absorption of photons to emit high NIR-to-BL luminescence rather than simultaneous two-photon absorption routes [28]. In a typical multiphoton activation process, these ions are excited by 980-nm NIR to an intermediate state. Subsequently, second photons pump them to an even higher excited state. Lower excitation densities in Tm^3+^/Yb^3+^ codoped nanomaterials usually result in 800-nm IR emission due to the energy transfer ^3^H_4_ → ^3^H_6_. The generation of blue emission requires a higher photon density to cause the energy transfer ^1^G_4_ → ^3^H_6_ [29]. The stepwise multiphoton upconversion fluorescence is characterized by enhanced blue emission at higher excitation power densities [29,30]. In this study, the as-obtained UP material presented a corresponding behavior since the intensity of blue emission appeared as a quadratic function of the NIR excitation irradiance (Figure 4b,c). A previous study indicated that the power density of NIR should exceed 355 mW to excite Yb^3+^ and sustain the energy necessary to transfer to codopants Tm^3+^ [29]. In this study, blue emission was detectable at a very low (74 mW) incident NIR power. This low excitation threshold could have contributed to sufficient Tm^3+^ and Yb^3+^ contents (each >8%) in our UP. Increasing the UP amounts did not proportionally enhance the BL emission intensity, which might be due to the light scattering of NIR through dense UP stacks (Figure 4c).

In the present study, the effects of UP particle size were assessed in two aspects: the emission intensity and polymerization of RBCs. The luminous intensity was highest in unground UP powder and decreased with the particle size. This result was attributed to the ball-milling preparations, which consequently induced surface defects on the particles to quench the fluorescence [29]. Additionally, the increased facets of the particles may disturb the light emission. In regard to the composite polymerization, 0.42-μm (6-h milling) UP showed the greatest microhardness values instead of the unground particles. The base composite Z100 was loaded with 0.01–3.5-μm inorganic fillers, and its d90 and d60 sizes were 1 and 0.3 μm, respectively [31]. The size distribution of 6-h milling UP particles (d50 = 0.42 μm) was quite similar to that of Z100, and, thus, facilitated their blending into composites that rendered homogenous distributions. For the future developments, new synthetic UP nanoparticles may work as good fillers for their proper size and strong upconversion fluorescence [32,33].

DC is an important parameter related to the final physical and mechanical properties of dental RBCs. Fourier transformation infrared spectroscopy (FTIR) [34] and Raman spectroscopy [35] are generally used to chemically quantify the monomer conversion. The FTIR coupled with Attenuated Total Reflectance (ATR) is specifically useful in the study of polymerization kinetics of RBCs [36]. However, the ATR-FTIR method only examines the surface of resin specimens or thin films. On the other hand, the microhardness tests show advantages in their simple techniques and ability to mutually assess DC and physical information [37,38,39]. Microhardness values have been proven to correspond with those spectroscopic methods and also correlate to mechanical properties including wear resistance, mechanical strengths, and stiffness of RBCs [40,41,42]. The depth profiles of microhardness are especially useful to investigate the extent of polymerization [37,43,44].

In this study, two UP composites showed enhanced microhardness with the combined BL-NIR curing. With these findings, the null hypothesis that the NIR-to-BL curing was comparable to conventional BL curing is rejected. It was supposed that the starting BL provided sufficient energy to initiate the radical chain reaction, and the following NIR upconversion luminescence homogeneously polymerized the composites. Incorporation of these exogenous UP fillers was not the primary cause of increased microhardness, since UP5 and UP10 showed comparable microhardness to Z100 under BL curing (Figure 6a and Figure 7a). Current dental restorative RBCs are highly loaded with silica or other inorganic fillers and their mechanical properties are predominantly related to the filler phases [45]. The base composite Z100 is almost saturated with the silica fillers (approximately 59 vol% and 79.4 wt%). The density of UP particles was lower than that of the silica fillers [15]. Incorporations of UP particles, in fact, reduced the fraction of inherent fillers; thus, the experimental composites attained lower Young’s modulus and hardness compared to commercial ones. The polymerization of the UP composites was subject to the interplay between the UP content and the duration of NIR irradiation. When the total irradiation time was within 100 s, UP5 showed significantly higher hardness values compared to UP10 and Z100 (Figure 6b and Figure 7b). Contrarily, UP10 exhibited superior surface and in-depth microhardness when the irradiation time exceeded 100 s (Figure 6b and Figure 7c). It is reasonable to assume that the UP10 had lower stiffness but was strengthened through extending NIR irradiation.

Different from previous studies, NIR alone did not sufficiently polymerize the UP-modified composite [15,23]. The differences mainly exist in the preparations of the experimental composites and the assessment method. In previous studies [15,23], UP particles were added into methacrylate monomer at loading rates up to 40 wt%. However, their UP-loaded composites exhibited inferior hardness compared to commercial products since these particles are less stiff than silica fillers [15]. In another study, by Stepuk et al. [23], the polymerization was assessed by manual needle penetration rather than a hardness test. With the present results, it is not recommended to replace the original filler systems in the restorative composites by UP particles.

Regarding the depth profiles of microhardness, the NIR upconversion regimen did not significantly increase the curing depths of resin composites (Figure 7). The inherent fillers of the Z100 composites may impair NIR transmission at the deep layer. A long NIR irradiation of 120 s did improve the microhardness of both UP5 and UP10 for the whole depths. Therefore, both increasing UP content and extending the NIR curing time are essential to improve the curing depth.

## 5. Conclusions

In this study, the NIR-to-BL upconverted photoluminescence was examined for its efficacy in curing dental RBCs. UP are suggested to work as adjunct fillers rather than constituent fillers in a dental composite. With the present results, 0.4-μm UP at the loading rate of either 5 wt% and 10 wt% were recommended in preparing the UP-modified composites. Under a combined BL-NIR curing protocol, these experimental composites showed enhanced polymerization with increased NIR duration and UP content. However, bare NIR irradiation insufficiently polymerized the composites. The NIR upconversion regimen could be a potential method in improving polymerization of dental composites with more refining works.

## Data Availability

The data presented in this study are available on request from the corresponding author.
